# Current Status of Fibroblast Growth Factor Receptor-Targeted Therapies in Breast Cancer

**DOI:** 10.3390/cells7070076

**Published:** 2018-07-15

**Authors:** Navid Sobhani, Anna Ianza, Alberto D’Angelo, Giandomenico Roviello, Fabiola Giudici, Marina Bortul, Fabrizio Zanconati, Cristina Bottin, Daniele Generali

**Affiliations:** 1 Department of Medical, Surgical & Health Sciences, University of Trieste, Cattinara Teaching Hospital, 34149 Trieste, Italy; fgiudici@units.it (F.G.); m.bortul@fmc.units.it (M.B.); fabrizio.zanconati@asuits.sanita.fvg.it (F.Z.); cris.bottin@gmail.com (C.B.); 2 Department of Medical, Surgery & Health Sciences, University of Trieste, 34129 Trieste, Italy; annaianzamiccoli@gmail.com; 3 Division of Medical Oncology, Department of Onco-Hematology, IRCCS-CROB, Referral Cancer Center of Basilicata, Rionero in Vulture (PZ), 85028 Rionero, Italy; giandomenicoroviello@gmail.com; 4 Department of Medical, Surgery and Health Sciences, University of Trieste, 34129 Trieste, Italy; Breast Cancer Unit and Translational Research Unit, ASST Cremona, Viale Concordia 1, C.A.P. 26100 Cremona, Italy

**Keywords:** fibroblast growth factor, fibroblast growth factor receptor, targeted treatments, breast cancer

## Abstract

Breast cancer (BC) is the most common malignancy and second only to lung cancer in terms of mortality in women. Despite the incredible progress made in this field, metastatic breast cancer has a poor prognosis. In an era of personalized medicine, there is an urgent need for better knowledge of the biology leading to the disease, which can lead to the design of increasingly accurate drugs against patients’ specific molecular aberrations. Among one of the actionable targets is the fibroblast growth factor receptor (FGFR) pathway, triggered by specific ligands. The Fibroblast Growth Factor Receptors/Fibroblast Growth Factors (FGFRs/FGFs) axis offers interesting molecular targets to be pursued in clinical development. This mini-review will focus on the current knowledge of FGFR mutations, which lead to tumor formation and summarizes the state-of-the-art therapeutic strategies for targeted treatments against the FGFRs/FGFs axis in the context of BC.

## 1. Introduction

### 1.1. The Biochemical Structure of the Receptor

Breast Cancer (BC) is the most common malignancy and second only to lung cancer in terms of mortality in women worldwide, with an estimated 268,670 new diagnoses and 41,400 deaths in 2018 in the US for both men and women [1]. With the advancement of personalized medicine, patients have been stratified on the basis of expression of actionable molecular targets. Among such actionable targets in BC is the fibroblast growth factor receptor (FGFR).

The FGFR family is characterized by four receptors, binding to 18 ligands called fibroblast growth factors (FGFs), employing heparin as a co-factor [2,3,4]. These receptors have pivotal roles in embryogenesis and metabolism [5,6], and play a critical role in the development of the skeletal system [7,8]. Fibroblast growth factors are secreted glycoproteins that are promptly sequestered by the extracellular matrix and at the cell membrane by heparan sulfate proteoglycans (HSPGs), which, in turn, make the FGF ligand-receptor interaction stable [9] by safeguarding FGFs from protease-mediated degradation [10]. Each ligand tethers the FGFRs with different specificity; some are promiscuous, such as FGF1, and bind to multiple receptors, while others, such as FGF7, tether only one receptor isoform [11]. FGFRs are a class of receptor tyrosine kinase (RTK) and are single-pass membrane proteins made of N-terminal extracellular (EC) domains with three immunoglobulin-like subdomains (D1, D2 and D3), a transmembrane (TM) domain with a single α-helix, and an intracellular (IC) region which includes tyrosine kinase motifs, a juxta-membrane domain and a carboxyl-terminal tail [12,13,14]. There is a total of seven signalling receptors, encoded by four *FGFR* genes, FGFR1-4 [15]. Furthermore, immunoglobulin-like subdomains D2 and D3 are necessary and sufficient for ligand binding, whereas the aminoterminal part of the receptor-including D1-has an auto-inhibitory function [16]. In addition, alternative splicing of the D3 extracellular fragment of FGFR1, 2 or 3 may encode isoforms that differ in relation to the specificity of ligand binding [17].

### 1.2. FGFR Signalling

**Figure 1:**
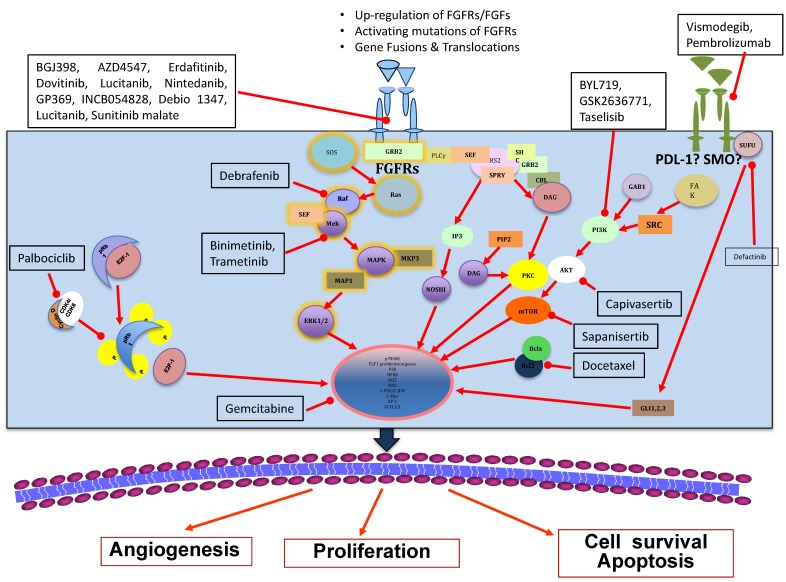
Current status of fibroblast growth factor receptor (FGFR) therapeutic strategies in breast cancer.

Since the discovery of RTKs around fifty years ago, the most widely accepted model of RTK transduction is the diffusion-based model, also known as the canonical model [18]. This model states that RTKs are monomers in need of a ligand for dimerization, thereby performing cross-phosphorylation and, consequently, activating one other [19]. After its activation, FGFRs transmit biochemical signals with lateral dimerization within the plasma membrane [20]. The dimerization of the receptor is a necessary step as it shortens the distance between the two tyrosine kinase domains, allowing them to cross-phosphorylate on tyrosine residues at the activation chain of the receptors [21,22]. These kinases’ triggering process tethers adaptors and phosphorylates proteins within the cytoplasm, triggering downstream signaling cascades [23,24]. Noteworthy among such adaptors is FGFR substrate 2 (FRS2), which, upon ligand binding and its association with the receptor, triggers downstream signaling with the activation of mitogen-activated protein kinase (MAPK) [25] and the phosphoinositide-3-kinase (PI3K)/AKT pathways [26]. Of note, FGFR signaling has also been found to be connected to phospholipase C-gamma (PLC-γ) in an FRS2-unrelated mode and activates protein kinase C (PKC) [27], which partially strengthens the MAPK pathway activation by phosphorylating RAF [28]. However, in relation to the cellular context, many other pathways might be activated by FGFRs, such as those involving a signal transducer and activator of transcription signaling and ribosomal protein S6 kinase 2 (RSK2) [29], as well as the p38 MAPK and Jun N-terminal kinase pathways [10,13,30,31]. Interestingly, all such related pathways are captivating targets to be explored in the context of clinical development of anti-cancer agents against the FGFs/FGFRs axis [32] (Figure 1).

### 1.3. The Control of FGFR Signalling

Regulation of FGF signaling is critical to ensure a balanced response to receptor stimulation. Unfortunately, the mechanism of attenuation is poorly understood and it is likely to vary depending on the cell type [17]. Nevertheless, the current understanding is that it takes place largely via a negative feedback mechanism, involving receptor internalization through ubiquitination [18,33] and induction of negative regulators, such as SEF, SPRY, SPRED 1 and 2 [19,34,35]. An additional level of control takes place in the form of receptor auto-inhibition [16,36]. For example, the electrostatic bonding between the acid box and the Heparane Sulfate (HS)-binding site creates an auto-inhibited closed conformation [6,37]. This process of auto-inhibition sustains FGF binding specificity to receptors [38].

## 2. FGFRs as Oncogenic Drivers

A long series of evidence is pointing towards the prospect that deregulated FGFRs can work as driving oncogenes in several types of tumor [10,39]. When an FGF receptor is deregulated, aberrant activation of downstream signalling results in mesenchymal, antiapoptotic and mitogenic responses in cells [40]. To date, several different FGFR pathway aberrations [41] have been discovered in cancer, and include: (i) Translocations of FGFR-fusion proteins with constitutive FGFR kinase activity [41]; (ii) gene amplification or post-transcriptional regulation resulting in high expression levels of the receptor protein [42]; (iii) upregulation of FGF in cancer cells, stromal cells or the extracellular matrix, showing paracrine/autocrine activation of the pathway [43]; (iv) alternative splicing of the genes encoding FGFR and FGFR isoform switching, which are alterations that modify ligand specificity, increasing the range of FGFs that can stimulate proliferation [44]; and (v) FGFR mutations that result in receptors that are constitutively active. According to Sarabipour et al. [45], in regards to the FGFR pathway aberration described in (v), FGFRs are capable of dimerize also without being triggered by ligands binding to them at physiological conditions, and these unbound dimers are stabilized via contact between the TM domains and IC domains [46]. Furthermore, unbound FGFR dimers are phosphorylated, providing an explanation for the fact that the overexpression of FGFR leads to cancer [4,47,48,49]. However, structural changes (induced by the ligand) that occur in the FGFR dimers in the plasma membrane and the ligand can control the structure of the TM domain, causing a switch to a specific conformation [50]. The resulting configuration of the TM dimers regulates the receptor activity [51]. Ultimately, the structural transformations in response to FGF1 and FGF2 are quite different, leading to different distances between the IC domains and a different level of phosphorylation of FGF1- and FGF2-bound dimers [52]. For this reason, there are several resulting active ligand-bound states for the FGF receptors [45]. In humans, many gain-of-function germline mutations in the *FGFR* genes cause skeletal dysplasias, and mutations in cancer are similar to those seen in hereditary diseases [53]. Intriguingly, these mutations are not limited to the kinase domain, but can cover the full length of the gene [54]. In particular, FGFR signalling in cancer shows a clear dependence with the context [55], resulting in aberrations differing according to tumor type [13,30,56,57]. For the purpose of this mini-review, the next section will focus on FGFR abnormalities that have been identified in breast cancer.

## 3. *FGFR* Genetic Alterations in Breast Cancer

The first documentation of the amplification of the *FGFR*s genes in human breast cancer relates back to the early 1990s [58]. A large series of studies since then have both confirmed the initial observation of the oncogenic potential of FGFRs and significantly expanded upon mechanisms by which the FGFs/FGFRs axis contributes to breast cancer formation. In addition to gene amplification, higher expression of ligands and receptors, mutations and single nucleotide polymorphisms have also been identified in FGFRs in BC patients’ samples, suggesting that more than one mechanism is involved in the aberrant activation of FGFRs [59].

The FGF/FGFR signaling pathway is frequently deregulated in human cancers. In breast cancer, *FGFR1* amplification is the most frequent genomic aberration, whereas the *FGFR2–4* gene amplifications and FGFR activating mutations are uncommon [4,60].

### 3.1. Amplification of FGFRs

About 14% of breast cancer patients bear mutations in the 8p11-12 chromosomal region, which is a site harboring the *FGFR1* gene locus [49,54,61]. Always in the context of BC, amplification of *FGFR1*- and/or 11q12-14, which is a chromosomal region containing *CCND1*, *FGF3*, *FGF4*, and *FGF19*, has been detected in 23% of hormone receptor-positive (HR+) BC, 27% Human Epidermal Growth Factor Receptor 2 (HER2)-positive BC, and 7% Triple Negative Breast Cancer (TNBC). These amplifications have also been shown to be a prognostic indicator for early relapses and poor patient outcomes [62,63,64,65,66]. As shown by in vitro studies, the expression of *FGFR1* is required for the survival of FGFR1-amplified BC cell lines, supporting the oncogenic role of *FGFR1* amplification. Using two cell lines with either *FGFR1* (MDA-MB-134) or *FGFR2* amplified (SUM52), Andrè et al. showed a reduction in both proliferation and tumor growth after treatment with anti-FGFR1 dovitinib (TKI1258) therapy [67]. The IC_50_ for cell growth inhibition was 190 nmol/L and 180 nmol/L. In negative controls that did not express either *FGFR1* or *FGFR2*, the IC_50_ values were more than 2000 nmol/L. Moreover, through the use of an in vivo mouse model with *FGFR1*-amplified BC primary xenograft (HBCx-3), the authors showed that the tumor regressed after treatment with 50 mg/kg of dovitinib, compared to mice treated with just a vehicle control (*p* < 0.001) [67]. Additionally, *FGFR1* amplification has been shown to drive resistance to endocrine therapy. In fact, Turner et al., through a viability assay, showed that the breast cancer cell lines MDA-MB-134 and SUM44, which overexpress *FGFR1* were resistant to 4-hydroxytamoxifen (4-OHT) [49]. Such resistance was reversed when the cells were treated with small interfering RNA against *FGFR1* (*siFGFR1*), suggesting that FGFR1 drives sensitivity to this type of therapy. Another proof of concept that such a mechanism of sensitivity to the drug was attributable to *FGFR1* comes from the fact that the addition of FGFR2 to siFGFR1-treated cell lines did not reverse the resistance to 4-OHT. Moreover, the authors proved that treatment with an FGFR1 inhibitor (PD173074) caused a loss of the cell lines’ ability to form colonies, suggesting that FGFR1 has a role in making those cancer cells capable of growing colonies, and therefore confirming its tumorigenic function in BC [49]. 

According to several studies, *FGFR2* amplifications-shown to occur in 4% of TNBC, as well as activating mutations of the receptor-have been associated with high sensitivity to FGFR inhibitors [67,68,69] and maintenance of tumor-initiating cells [68].

### 3.2. FGFRs Activating Mutations

In the context of FGFR-driven tumor formation, and although *FGFR* gene amplifications are the most common type of alterations leading to BC, there is evidence that the activating mutations also have an oncogenic role in this type of cancer [4]. Activating FGFR mutations may result in aberrant FGFR signaling through different mechanisms, including: (i) Constitutive dimerization of the receptors; (ii) enhancement of the kinase domain’s activity; and (iii) alteration in affinity for FGF ligands. In fact, the most commonly occurring oncogenic FGFR aberrations in BC are the following: *FGFR1* translocation [70]; *FGFR1* amplification (10–15%) [49,62,63,64,65,66,67], which has shown transforming potential in several in vivo models, conferring sensitivity to FGFR inhibitors [65] and an ability to drive resistance to endocrine therapy [49], as previously described; *FGFR2* translocation, which in preclinical models exhibited transforming potential and sensitivity to FGFR inhibitors [70]; and FGFR2 amplifications (4%, [68]), which in preclinical models conferred resistance to FGFR inhibitors [67,68,69]. In different solid tumors, including breast cancer, various studies in the literature have described FGFRs activating mutations:

FGFR1: Two point mutations (K656E and N546) have been observed in vitro affecting the intracellular domain of the receptor, and were therefore operating as activating mutations [71,72].

FGFR2: In the Catalog Of Somatic Mutations in Cancer (COSMIC) database, 12 mutations have been reported, but only seven of them are activating mutations (missense mutations) of the extracellular domain, with the most common ones being P253R, N549K and S253R [54].

FGFR3: In the COSMIC database, 13 different point mutations have been described, with S249C being the most common. The most frequent activating mutations of FGFR3 affect either the transmembrane (A391E, G370C, G380R, Y373C, S371C) or the extracellular (R248C, S249C) domains of the protein. Rarer mutations are those in the kinase domain (N540S, K650E, K650M, K650N, K650Q, and K650T) [54,73].

FGFR4: There are only four FGFR4 activating mutations occurring in the kinase domain. Two of them (E550 and K535) cause auto-phosphorylation, and therefore induce constitutive activation of the receptor [54,74].

Some of these mutations have been associated with an increased risk of developing breast cancer. Genome-Wide-Association-Studies (GWAS) from several independent research groups showed how Single Nucleotide Polymorphisms (SNPs) on intron 2 of FGFR2 is a risk factor associated with disease [75,76,77,78]. In fact, Easton et al., using their GWAS composed of 4398 BC cases and 4316 controls, investigated commonly known SNPs to find risk factors associated with the disease [75]. The authors identified SNPs in five new loci that exhibited strong and consistent association with breast cancer (*p* < 10^−7^). Among these loci there was a FGFR2 whose oncogenic role in BC had already been consolidated in the literature [75]. Accordingly, Stacey et al., in their GWAS made up of 6145 BC cases and 33016 controls, identified two SNPs (rs4415084 and rs1094179) on 5p12, which conferred a risk in developing BC, especially in Estrogen Receptor Positive (ER) + BC (*p* = 1.3 × 10^−17^) [76]. By the use of gene expression microarray data, Meyer et al. showed that there is a trend of increasing FGFR2 expression in rare homozygotes [77]. Moreover, Meyer et al. demonstrated by Real-Time PCR (RT-PCR) that there is a different trend between the FGFR2 rare and common homozygotes (Wilcox *p*-value of 0.028), and proved that Oct-1/Runx2 binding site is probably the dominant determinant for such differential expression [77]. According to Easton et al. [75] and Stacey et al. [76], the GWAS of Hunter et al. [78] identified alleles in FGFR2 associated with a higher risk of sporadic post-menopausal BC. Their study investigated 528,173 SNPs in 1145 postmenopausal women of European ancestry with invasive BC and 1142 controls [78]. The authors identified several genomic locations as potentially associated with BC, and four of the ones with the most significant *p*-values-r1219648, rs2420946, rs11200014 and rs2981579-were located on intron 2 of FGFR2.

Although GWAS from several groups has confirmed that a germ-line polymorphism in intron 2 of FGFR2 is associated with BC susceptibility [75,76,77,78], emphasizing the relevance of FGFR2 in BC development, little is known about the mechanism by which FGFR2 functions as a risk factor leading to BC. A plausible explanation comes from the work of Kim et al., which showed that FGFR2 promotes breast cancer tumorigenicity by maintaining tumor-initiating cells (TICs) [68]. As a matter of fact, the authors revealed in their model of BC TICs-isolated through flow-cytometer with CD29^high^ CD24^+^ markers-that there were several markedly upregulated genes compared to non-TICs. The genes that exhibited significantly higher mRNA expression levels were GABRA4, FGFR2 and FOXA1. The group then also proved that the FGFR2 protein levels were higher in TICs. Furthermore, their in vivo results demonstrated that down-regulation of FGFR2 by short hairpin RNA (shRNA) substantially reduced (64 to 70%) the TICs subpopulation (CD29^high^ CD24^+^). Intriguingly, shFGFR2 significantly increased (65 to 67%) the subpopulation of non-TIC cells (CD29^low^ CD24^−^). These results suggest that FGFR2 causes a decrease of TICs and an increase of non-TICs [68]. Moreover, the authors showed that FGFR2 in shFGFR2-treated mice resulted in a considerable increase of bipotent precursor-like cells (K18+K14+), suggesting that FGFR2 rescued the bipotent capacity driven by the *FGFR2* knockdown. Therefore, inhibiting FGFR2 could be a valid strategy to destroy those TICs populations in BC. Additionally, Kim et al., in an in vivo mice model, found that treatment with FGFR2-inhibitor (TKI258) suppressed tumor growth. Such a growth inhibition was accompanied by a significantly reduced phosphorylation of FGFR2 and Erk1/2, suggesting that such an inhibition was dependent on FGFR2 activation and its targets [68]. Guagnano et al. screened 541 cancer cell lines—including BC cell lines-for “*FGFR* genetic alterations”, and investigated the sensitivity of the cells to an anti-FGFR inhibitor (NVP-BGJ398). They considered nine distinct types of *FGFR* genetic alterations already established in the literature: FGFR1-4 copy number gains; FGFR1-3 activating mutations; and *FGFR1* or *FGFR2* chromosomal translocations. Their compound NVP-BGJ398 proved to be a strong multi-kinase inhibitor targeting FGFR1-4 and the Vascular Endothelial Growth Factor Receptor (VEGFR) 2. Finally, the research group showed that such *FGFR* genetic alterations are considered a top predictor of the response to NVP-BGJ398 [69]. In a small study employing comprehensive molecular analyses of 13 lobular breast carcinomas, Reis-Filho et al. demonstrated that a high level of gains was detected at the chromosomal position 8p12-p11.2 in six of their primary cases [65]. Furthermore, through siRNA and a small-molecule inhibitor of FGFR1 (SU5402), they proved that inhibition of FGFR1 was capable of blocking survival of the ductal carcinoma cell line MDA-MB-134 [65]. Therefore, the analyses of different research groups are supportive of the fact that inhibition of the FGFR/FGF axis could be a valid approach for further investigation in large and randomized clinical trials. According to more recent studies based on Next Generation Sequencing (NGS), the levels of FGFR3 and FGFR4 were very low in BC. In fact, in a NGS study by Helsten et al., made up of 4853 solid tumors (including 522 BCs), the authors proved that the amplification of the FGFR3 and FGFR4 is expressed in less than 1% and 2% of BC patients, respectively [54]. On the other hand, in a previous study based on RT-PCR and made of 10 tumor cell lines and 103 breast-tumor samples, FGFR4 was expressed in up to 32% of patients with BC while FGFR3 was not detected [79].

### 3.3. Gene Fusions of FGFRs

Gene fusion involves the joining of two different genes, either via a translocation or an inversion. It represent 8% of FGFR aberrations [4,54]. There are at least 11 fusion partners identified for FGFR1. Such fusions include ZNF198, BCR and FOP. The most commonly occurring *FGFR* genes with this kind of alteration are the FGFR2 and the FGFR3. The majority of gene fusions have been identified in patients with myeloproliferative disorder stem cell leukemia/lymphoma syndrome. Gene fusions with the *TACC3* gene, resulting in a FGFR3-TACC fusion protein, lead to a constitutive activation of the receptor [20,80]. As for breast cancer, *FGFR1–3* gene fusions have been observed to occur with multiple partners (i.e., TACC1, TACC2, TACC3, BAIAP2L1, BICC1, NPM1, PPAPDC1A, AFF3, SLC45A3 and AHCYL1) [54,70,81].

## 4. Anti-FGFR Therapies

The relevance of the FGFR/FGF pathway in the development and progression of BC justifies the growing interest in developing new, targeted therapies for this pathway [82]. Small inhibitors of FGFR tyrosine kinase, selective or nonselective, are under clinical evaluation, although mainly in the early stages of trials [83]. Efforts are being made to increase the selectivity to the intracellular ATP-binding domain of the receptor to minimize the toxicity [30]. BGJ398 (infigratinib), a pan-FGFR inhibitor, is currently under evaluation as a single agent to establish the maximum tolerated dose (MTD) (NCT01004224) [84]. Moreover, another phase I trial (NCT01928459) was conducted in order to determine the MTD for BGJ398 with BYL719 for the treatment of solid tumors bearing *FGFR 1–3* alterations and *PIK3CA* mutations. AZD4547 is another TKI that has shown strong activity against FGFR-3, yet weaker activity against FGFR4. Its safety and effectiveness is under evaluation in ER+ patients harboring *FGFR1* polisomy or gene amplification after progression to endocrine-based therapies (NCT01791985). Another phase I study (NCT03238196) has been conducted for ER+ HER2- metastatic breast cancer (MBC) patients in order to evaluate Erdafitinib, which is an orally administered FGFR inhibitor, in combination with anti-CDK4/6 palbociclib and anti-HR fulvestrant. 

The development of non-selective TKI-targeting FGFRs has recently been shown to be very successful in preclinical studies [6]. Some of these inhibitors have passed the phase I trial with encouraging results in terms of safety and tolerability. TKI258 (dovitinib) is effective against VEGFR1-3, FGFR1-3 and PDGFR [37], and was under evaluation in combination with fulvestrant for the treatment of HER2 negative metastatic breast cancer, however this was terminated due to slow and low enrollment (NCT01528345).

E3810 (lucitanib), a drug that inhibits VEGFR1-3, FGFR1, colony stimulating factor 1 receptor (CSF1R), and FGFR2 has been administered as a single agent in two phase II trials in MBC patients with or without *FGFR1* amplification; one phase II study (NCT02202746) is ongoing. A phase I study is currently evaluating the safety and tolerability of the combination of letrozole and nindetanib, a triple kinase inhibitor (VEGFR, PDGFR, and FGFR), in postmenopausal women with ER+ MBC (NCT02619162).

Other strategies used to inhibit the FGFR/FGF axis are under investigation [85]. Similar to the development of antibodies against HER2+ isoforms, antibodies against FGFR isoforms represent a valid therapeutic strategy to intervene in BC. As a matter of fact, GP369 recognizes FGFR-IIIb isoform and has exhibited good results in blocking breast cancer cell line proliferation [86]. Such positive preliminary results warrant further research. Lastly, another approach against the FGFR/FGF axis concerns the use of inhibitors of FGF ligands. Long pentraxin-3 (PTX3) is an inhibitor of various FGFR ligands, among them FGF2 and FGF8b, which have both been found to be implicated in breast cancer development [87]. FP-1039 is a recently developed ligand-trap in which a ligand-binding domain of FGFR1 is fused to an Ig-Fc domain. This compound showed promising activity in vitro and passed a phase I clinical trial (NCT00687505) for solid tumors, including breast cancer [88]. Of note, FGFR may play a role in the development of resistance to anti-VEGFR therapy. Therefore, a proposed strategy is the use of small molecules targeting both receptors [89]. Additionally, several studies have suggested that inhibition of FGFR activation may lead to synergic activity with endocrine-based therapies and anti-ErbB therapies. For this reason, it would be interesting to consider the combination of anti-FGFR therapies with other already established treatments for breast cancer, targeting other pathways in order to obtain an increased effect while developing more powerful molecules to better treat this disease [90]. Table 1 summarizes ongoing clinical trials testing anti-FGFRs therapies in breast cancer. These results are certainly relevant, but a deeper understanding of the FGFR action in the promotion of breast cancer and its connection with other already established pathways are surely needed.

## 5. Discussion

FGFR is an already established BC oncogenic driver involved in various mechanisms leading to the formation of vessels, tumor growth and avoidance of apoptosis. Various genetic alterations of FGFR have been associated with BC, and thus therapeutic strategies have been implemented in order to inhibit FGFRs. In fact, several anti-FGF/FGFR therapies have been tested at phase I and II clinical trials. Among them are the FGFR inhibitors erdafitinib and nindetanib, a pan-FGFR inhibitor infigratinib, and FGFR1–3 inhibitors AZD4547 and dovitinib. It is worth noting that *FGFR1* amplification is the most frequent genomic aberration, whereas the *FGFR2–4* gene amplifications and FGFR activating mutations are uncommon. Therefore, for future therapeutic strategies involving FGFRs in BC, the FGFR1 should be considered as a primary target to be predominantly pursued as it is the most commonly altered *FGFR* gene currently in this context. The role of anti-FGFR therapies should be tested in combination with other molecules targeting downstream molecules of the same pathway, FGFs, and other tyrosine kinase cell membrane receptors like EGF, PDGF, VEGF, CCK, AXL, ROS, RET, RYK, TIE, LMR and HGF. Through the stratification of patients in groups on the basis of specific molecular alterations and evaluation of increasingly accurate predictive biomarkers, it becomes easier to choose which combination of therapies could be most beneficial for the patients. Moreover, in an era where immunotherapy is at the front-line of innovation, it would be interesting to test combinations of anti-FGFR or anti-FGF therapies with specific immune stimulating molecules-like with checkpoint inhibitors-in order to improve survival and quality of life of BC patients with novel and increasingly accurate therapeutic strategies.

## Figures and Tables

**Table 1:** Selected ongoing trials with FGFR inhibitors in breast cancer.

Clinical Trial Identifier	Study Design	Intervention/s	Setting	Primary Endpoint	Phase	Status
NCT03238196	32 Participants, Non-Randomized, Open label	Fulvestrant + palbociclib + erdafitinib as an escalation (Arm A: 4–8 mg once daily for erdafitinib, 125 mg once every 21 days followed by 1 week of rest (without taking the drug) and 500 mg once daily for erdafitinib) or the same combination of drugs as an expansion (Arm A: 4–8 mg once daily for erdafitinib, 125 mg once every 21 days followed by 1 week of rest (without taking the drug) and 500 mg once daily for erdafitinib).	Second line	Safety and Tolerability	1	Recruiting
NCT02465060	6452 participants, Non-Randomized, Parallel assignment, Open Label	Adavosertib, afatinib, binimetinib, capivasertib, crizotinib, dabrafenib, dasatinib, defactinib, AZD4547, larotrectinib, nivolumab, osimertinib, palbociclib, pertuzumab, GSK2636771, sapanisertib, sunitinib malate, taselisib, trametinib, trastuzumab, trastuzumab emtansine, vismodegib	Second line	OR	2	Recruiting
NCT02202746	178 participants, Parallel Assignment, Open label	Lucitanib in patients with FGFR1-amplified or 11q-amplified (Arm A: 10 mg once daily), and in patients with FGFR1- non amplified and 11q non-amplified (Arm B: 10 mg once daily)	Second Line	PFS	2	Active, not recruiting
NCT01004224	208 participants, Single group assignment, Non-Randomized, Open label	BGJ398 (dose escalation)	Second line	MTD	1	Active, not recruiting
NCT01791985	56 participants, Single group assignment, Open label	Anastrazole (1 mg daily), letrozole (2.5 mg once daily) and AZD4547 (80 mg twice daily)	Second line	Safety and Tolerability	1 & 2	Active, not recruiting
NCT02619162	22 participants, Single group assignment, Open label	Letrozole (2.5 mg) with nintedanib (100–150 mg)	Second line	DLT	1	Recruiting
NCT03344536	55 participants, Single group assignment, Open label	Fulvestrant (500 mg 1, 15, 29 and every 28 days (+/− 3 days) thereafter) and Debio 1347 (dose escalation, administered once daily).	Second line maximum for phase II; phase I could have received more than one prior treatment	DLT	1 & 2	Recruiting
NCT02393248	280 participants, Single group assignment, Open label	Combination therapy: Gemcitabine + Cisplatin + INCB054828; Pembrolizumab + INCB054828; Docetaxel + INCB054828; Trastuzumab + INCB054828.	Second line	MTD	1 & 2	Recruiting

Abbreviations: Progression Free Survival, PFS; Objective Response, OR; Dose Limiting Toxicity, DLT; Maximum Tolerated Dose, MTD.

## Abbreviations

BC	Breast Cancer
FGF	Fibroblast Growth Factor
FGFR	Fibroblast Growth Factor Receptor
VEGFR	Vascular Endothelial Growth Factor
HPSGs	heparan sulfate proteoglycans
EC	N-terminal extracellular
TM	transmembrane
IC	intracellular
PI3K	phosphoinositide-3-kinase
MAPK	mitogen-activated protein kinase
PKC	activates protein kinase C
4-OHT	4-hydroxytamoxifen
TICs	maintaining tumor-initiating cells
GWAS	Genome-Wide-Association-Studies
